# Impact of Periprocedural Risk Predictors on Long-Term Outcomes in Patients with Diabetes Undergoing Coronary Artery Bypass Grafting

**DOI:** 10.3390/medicina62010071

**Published:** 2025-12-29

**Authors:** Aleksander Dokollari, Serge Sicouri, Basel Ramlawi, MaryAnn Wertan, Francis P. Sutter

**Affiliations:** 1 Department of Cardiac Surgery Research, Lankenau Institute for Medical Research, Main Line Health, 100 East Lancaster Avenue, Suite 215, Wynnewood, PA 19096, USA; 2 Cardiac Surgery Division, St. Boniface Hospital, University of Manitoba, Winnipeg, MB R2H 2A6, Canada

**Keywords:** coronary artery bypass grafting, diabetes mellitus, long-term outcomes, coronary artery disease, risk predictors

## Abstract

*Background and Objectives:* In this study, we aim to analyze the impact of risk predictors on long-term outcomes in patients with diabetes undergoing isolated coronary artery bypass grafting (CABG). *Materials and Methods:* All consecutive patients undergoing isolated CABG between May 2005 and June 2021 were included in the study. Patients with and without diabetes were compared for baseline demographics and pre-operative characteristics. A propensity-matched analysis was used to compare the two groups. The primary outcome was long-term incidence of all-cause death. *Results:* Of a total of 4871 patients, propensity matching identified 1589 pairs of patients with and without diabetes that were included in the current study. Median follow-up was 5.8 years. All-cause death was recorded in 215/1589 (13.5%) vs. 169/1589 (10.6%) patients with and without diabetes, respectively (HR 1.3, *p* = 0.013). MACCE was also significantly higher in diabetic patients (HR 1.3, *p* = 0.049). Diabetes mellitus was identified as one of the independent predictors for all-cause mortality (HR 1.4, CI 1.2, 1.7) and MACCE (HR 1.2, CI 1.0, 1.3). Chronic obstructive pulmonary disease, peripheral vascular disease, and serum creatinine levels >2.0 mg/dL were found to be the only predictors of all-cause mortality in both diabetic and non-diabetic patient groups, when individually analyzed. *Conclusions:* Patients with diabetes undergoing isolated CABG had a significantly higher incidence of late all-cause death and MACCE compared to those without diabetes. The presence of diabetes mellitus predicts poorer long-term outcomes following CABG.

## 1. Introduction

Diabetes in patients with coronary artery disease (CAD) undergoing coronary artery bypass grafting (CABG) has been widely investigated. The risk of future major cardiovascular and cerebrovascular events (MACCE) is significantly higher in CAD patients with diabetes compared to those without diabetes [1,2]. Revascularization strategies are also known to differ for those with more extensive disease, including multivessel CAD [3,4]. In this context, the Revascularization in Diabetes Study (CARDia) and a subgroup analysis of the synergy between percutaneous coronary intervention (PCI) with TAXUS and Cardiac Surgery (SYNTAX) study showed an increased rate of MACCE at 12 months for patients with multivessel disease who underwent PCI with drug-eluting stents, in comparison to CABG [1,2]. In addition, the Future Revascularization Evaluation in Patients with Diabetes Mellitus: Optimal Management of Multivessel Disease (FREEDOM) clinical trial [3] showed that CABG is associated with reduced long-term mortality compared to PCI in patients with diabetes and multivessel CAD [3].

However, controversies remain over the impact of risk predictors on long-term prognosis in patients with diabetes undergoing isolated CABG [4]. In this context, the impact of diabetes on short-term mortality and morbidity in patients undergoing CABG is unclear. With a few exceptions [5], studies are consistent in documenting a 50% to 90% increase in long-term mortality rates in diabetics [6,7,8]. Another clinical study found that diabetic patients did not show higher rates of mortality in comparison with non-diabetic patients, but had more total neurological complications, more renal complications, a higher re-opening rate, a more prolonged ICU stay, and they needed more blood transfusions [9]. Our study’s main goal is to understand the differences in outcomes in diabetic vs. non-diabetic patients and analyze what drives these differences. This way, we can understand what risk factors can be reversed and which are innate.

## 2. Patients and Methods

### 2.1. Patients

Our study included all consecutive patients who underwent isolated CABG between May 2005 and June 2021 at Lankenau Heart Institute (Lankenau Medical Center, Wynnewood, PA, USA). The study protocol was approved by the Main Line Health Hospitals Institutional Review Board (IRB 45CFR164.512): approval date 10 December 2021. Patients with concomitant surgical procedures were excluded from the analysis. Patients were identified via operation codes in a digital operation registry. Clinical data was derived from medical records that were populated prospectively. The in-hospital outcomes were acquired from the charts and death certificates filled out by the responsible doctor.

### 2.2. Follow-Up

Follow-up was performed at our outpatient clinic and from the hospital registry. All patients had at least one follow-up time point available.

### 2.3. Group Analysis

The patients were divided into 2 groups based on the presence or absence of pre-operative diabetes to compare demographics, pre-operative characteristics, early results, and late outcomes following isolated CABG between diabetic and non-diabetic patients. Diabetic patients were defined as those with a history of diabetes, regardless of duration of disease or need for anti-diabetic medications [10]. During the study period, eleven surgeons performed CABG procedures in our institution.

### 2.4. Primary and Secondary Objectives and Definitions

The primary outcome was the long-term incidence of all-cause death and a component of the secondary outcomes (MACCE). The secondary outcome was the incidence of MACCE, which included all-cause death, myocardial infarction (MI), stroke, and reoperation. Other clinical definitions are included in. Patients’ pre-operative characteristics included in the statistical analysis are described in Table S1. Creatinine clearance was calculated according to the Cockcroft–Gault equation.

### 2.5. Statistical Analysis

Propensity score matching was used via multiple logistic regression with diabetic status as the dependent variable and inclusion of all demographic and pre-operative parameters into the model to reduce allocation bias and confounding parameters. For the propensity matching, a 1:1 greedy nearest neighbor with no replacement match and a caliper width of 0.2 produced 1589 patients in each group. The success of matching was assessed by computing the percent bias (similar to standardized mean difference) of each covariate, with a cut-off of 10% to denote acceptable balance. Matched samples were compared using McNemar’s test, and marginal homogeneity tests were used for categorical variables, and matched-pairs *t*-tests and signed rank tests for continuous variables. Estimated weights were incorporated into a Cox-proportional hazards regression model, then into two different Cox-proportional hazards regression models for both diabetic and non-diabetic groups, analyzing primary and secondary endpoints. Both the unmatched and matched Cox regression models were adjusted for potential confounding variables.

To illustrate the effect of diabetes on long-term survival, cumulative survival and MACCE curves were constructed and compared by the log-rank test. Adjusted survival functions for these interactions were plotted using Stata’s curve command. A sensitivity analysis was performed using diabetes status as a time-dependent variable in a series of covariate-adjusted Cox-proportional hazards regression models for all-cause mortality and the falsification endpoints using variables from the propensity model as covariates. This analysis allowed the inclusion of patients who died within the first 30 days following surgery and was performed to account for possible immortal time bias. Additional long-term statistical analysis is depicted in. We found risk predictors for all-cause death, MACCE, stroke, MI, and reoperation for the entire population. We then found risk predictors for all-cause death, MACCE, stroke, MI, and reoperation separately for diabetic and non-diabetic patients.

All analyses were performed in Stata 17.0 (StataCorp, LLC., College Station, TX, USA), and 95% confidence intervals and *p*-values are reported, with a *p*-value < 0.05 being considered significant.

## 3. Results

A total of 4871 consecutive patients who underwent isolated CABG were divided into 2 groups: 2019 patients with diabetes and 2852 patients without diabetes (Table 1).

### 3.1. Pre-Operative Characteristics

Following propensity matching, each group contained 1589 patients, with the maximum percentage bias being 3.3% among the pre-operative patient characteristics between groups, thereby indicating a very good match (Table 1).

### 3.2. Intra-Operative Characteristics

No significant differences were observed between groups in intra-operative parameters (Table 2), and 20% of patients in both groups underwent total arterial revascularization.

### 3.3. Post-Operative Outcomes

Post-operative outcomes demonstrated that patients with diabetes had higher rates of cryoprecipitate and platelet transfusions (*p* < 0.05), stroke (*p* = 0.01), and superficial wound infections (*p* = 0.035), as well as longer hospital length of stay (LOS) (*p* = 0.0005) (Table 3).

### 3.4. Follow-Up

The median follow-up survival of patients in the diabetic and non-diabetic groups was 3.9 (1.1–7.3) and 3.5 years (1.1–7.2) (*p* = 0.534), respectively (Table 4).

**Figure 1:**
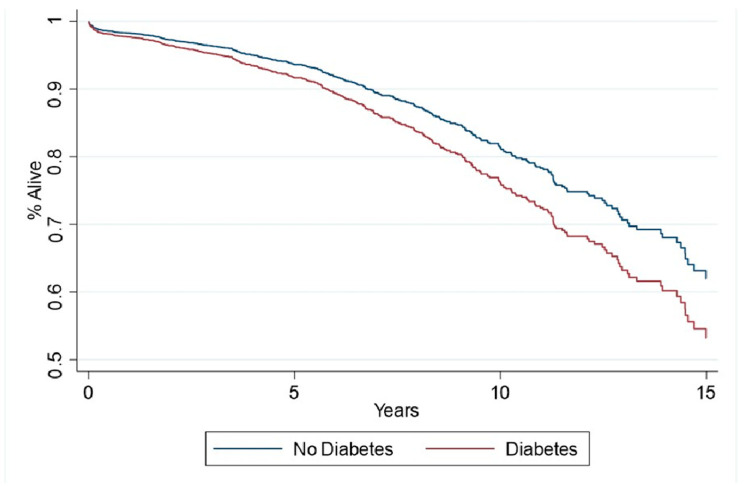
Cumulative Incidence Analysis of Long-Term Survival in Matched Patients.

The cumulative incidence analysis of long-term outcomes revealed a death rate of (215/1589 [13.5%] vs. 169/1589 [10.6%]; HR 1.3 [1.04, 1.6]; *p* = 0.013) in diabetic vs. non-diabetic patients (Figure 1). MACCE rates were significantly higher in patients with diabetes compared to those without diabetes (264/1589 [16.6%] vs. 228/1589 [14.4%]; HR 1.3 [1.1, 1.4]; *p* = 0.049) (Table 4). Incidence of stroke (*p* = 0.903), spontaneous MI (*p* = 0.431), reoperation (*p* = 0.699), and recurrent angina (*p* = 0.33) were not significantly different between groups. Sensitivity analysis revealed a higher overall mortality at 1- (44 [2.8%] vs. 29 [1.8%]; HR 1.7 [1.05, 2.7], *p* = 0.076); 2- (63 [4.0%] vs. 41 [2.6%] patients; HR 1.7 [1.1, 2.5], *p* = 0.028); 5- (111 [7.0%] vs. 79 [5.0%] patients; HR 1.5 [1.1, 2.0], *p* = 0.017); and 10-year (181 [11.6%] vs. 143 [9.1] patients; HR 1.3 [1.04, 1.6], *p* = 0.023) follow-up periods after isolated CABG in patients with diabetes compared to those without diabetes. Similarly, MACCE rates were also higher in diabetic compared to non-diabetic patients at 1 (54 [3.4%] vs. 33 [2.1%] patients; HR 1.8 [1.2, 3.0], *p* = 0.022); 2 (75 [4.7%] vs. 50 [3.2%] patients; HR 1.6 [1.1, 2.4], *p* = 0.023); and 5 years (140 [8.8%] vs. 112 [7.1%] patients; HR 1.3 [1.05, 1.8], *p* = 0.046) following surgical revascularization, respectively. However, at 10 years, the difference in MACCE rates was not significant between groups (225 [14.2%] vs. 196 [12.3%] patients; HR 1.2 [0.99, 1.5], *p* = 0.129).

### 3.5. Risk-Predictor Analysis

Cox regression analysis of the entire matched population identified diabetes mellitus as an independent predictor of all-cause mortality among other factors such as age > 75 years, BMI > 30 kg/m^2^, serum creatinine levels > 1.2 mg/dL, prior MI, and PVD (Table 5).

When individually analyzed, the characteristics of age > 75 years, prior heart valve surgery, prior MI, and atrial fibrillation predicted all-cause death in non-diabetics, but not in diabetics, whereas serum creatinine levels between 1.2 and 2.0 mg/dL predicted late mortality only in diabetic patients. COPD, PVD, and serum creatinine levels > 2.0 mg/dL were predictors in both diabetic and non-diabetic patients. Independent predictors for MACCE, spontaneous MI, stroke, and reoperations are depicted in Table 5.

## 4. Discussion

The principal findings of the current study reveal the following:

(I) Patients with diabetes are at a higher risk of death and occurrence of MACCE compared to those without diabetes following isolated CABG surgery (Table 4);
(II) The presence of diabetes mellitus was an independent risk factor for late mortality and MACCE;
(III) COPD, PVD, and serum creatinine levels > 2 mg/dL predicted all-cause mortality and MACCE in patients with and without diabetes mellitus.

As mentioned in the Introduction section, there is an incongruence in the medical literature regarding all-cause mortality outcomes in patients with diabetes vs. those without undergoing CABG. This study clarified that all-cause mortality is higher in patients with diabetes compared to non-diabetic patients. Most importantly, the study found out what the risk factors are that influence these outcomes, and which are those that can be reversed.

### 4.1. All-Cause Death

Our study revealed that patients with diabetes undergoing isolated CABG have lower survival and a greater risk of MACCE compared to those without diabetes. Our findings are similar to those reported by the SYNTAXES study, which demonstrated a higher 10-year mortality of 34.5% versus 21.4% amongst diabetic and non-diabetic patients, respectively (HR 1.65, CI 1.19–2.28, *p*-value 0.003). Likewise, the BARI trial also recorded a better 10-year survival in patients without treated diabetes in comparison to those with diabetes (77.3% vs. 57.9%) [10]. The CABG arm of the FREEDOM trial [3], which only included patients with diabetes, reported an all-cause mortality of 9.9% and 18.7% at 5- and 8-year follow-up after CABG.

The all-cause mortality in our study was 13.7% and 25.1% at 5 and 10 years following CABG in diabetic patients. The higher mortality rate can be attributed to the higher mean age, greater prevalence of previous MI, and a higher proportion of diabetic patients with left ventricular dysfunction in our series. On the contrary, our long-term outcomes were better than those reported by the SYNTAXES and BARI trials, despite patients being older in our series.

### 4.2. Incidence of MACCE

The Coronary Revascularization Demonstrating Outcome Study in Kyoto Percutaneous Coronary Intervention/Coronary Artery Bypass Graft (CREDO-KYOTO) registry, which is a large-scale pooled registry of consecutive patients undergoing their first coronary revascularization with PCI or CABG in Japan, recently reported that diabetic patients had worse cardiovascular outcomes up to 5 years following PCI or CABG [11,12,13]. The higher MACCE (all-cause death, MI, and stroke) rates in diabetic patients were chiefly driven by higher all-cause mortality, which was primarily caused by a greater risk of cardiovascular death. Tam and colleagues reported an overall MACCE incidence of 30.4% in patients with diabetes undergoing CABG in Ontario, Canada [14]. Likewise, Farkouh and colleagues reported an overall incidence of 18.7% of MACCE from 30 days to 5 years follow-up in patients with diabetes [15]. Our MACCE results are similar to those reported in the aforementioned studies, thereby emphasizing the negative impact diabetes has as a chronic disease on longitudinal outcomes following CABG in patients with CAD. Furthermore, our study identified and compared risk predictors influencing all-cause mortality and MACCE outcomes in patients with and without diabetes.

### 4.3. Predictors for Late All-Cause Death and MACCE in Patients with Diabetes

The above-mentioned report of the CREDO-KYOTO registry further identified a significant interaction between adjusted risk of diabetes relative to non-diabetes, especially in younger patients, whereas no significant interaction was observed between the diabetic status of patients and other subgroups such as sex, mode of revascularization, and clinical presentation of MI [13]. Another study from Brazil comparing outcomes following CABG between diabetic and nondiabetic octogenarians did not find any difference in in-hospital mortality and morbidity [16]. Our study echoes these findings, as age > 75 was found to be a significant predictor of all-cause mortality in non-diabetic patients (HR 1.04, CI:1.01–1.04), but not in those with diabetes (HR 0.98, CI:0.98–0.99). Gender was not found to be a predictor, irrespective of the diabetic status of patients. In addition, only a few publications analyzed the impact of risk predictors on long-term survival and MACCE. In this context, Straten and colleagues [17,18] reported that risk predictors for long-term all-cause death included age, COPD, low creatinine clearance, EF less than 35%, PVD, previous cardiac surgery, female sex, emergency operation, and hypertension. Furthermore, complications such as perioperative myocardial infarction, perioperative use of intraaortic balloon pump (IABP), and re-exploration were identified as risk factors for late mortality. Thourani and colleagues also reported that predictors impacting cardiovascular repeat events in patients with diabetes included age, urgent/emergent operation, EF, female sex, and hypertension, and these risk factors impacted long-term prognosis [19].

### 4.4. Incidence of Nonfatal Stroke

At the 30-day follow-up, our study evidenced a stroke incidence of 1.1% while the FREEDOM trial reported an incidence of 1.8% of stroke, and the SYNTAX II trial showed a stroke rate of 2.2%. We hypothesize that the low incidence of nonfatal stroke rate is mostly related to the no-touch aortic technique performed at Lankenau, where almost 85% isolated CABG procedures are performed off-pump. Five-year outcomes of the FREEDOM trial reported a stroke rate as low as 5.2% in diabetic patients undergoing CABG [15]. In addition, 5-year outcomes from the SYNTAX II clinical trial evidenced an overall stroke rate of 3.7%, while most patients (1.6%) had an ischemic stroke [20]. This analysis reported a stroke rate as low as 2% at the 5-year follow-up and 3.7% at the 10-year follow-up.

### 4.5. Incidence of Myocardial Infarction

Thirty-day clinical outcomes from the FREEDOM clinical trial and the SYNTAX II clinical trial evidenced an overall incidence of MI of 1.7% and 3.3%, respectively. In addition, Thourani and colleagues [21] and Straten and colleagues [17] reported an overall incidence of 30-day MI of 1.8% and 2.1%. In our study, the overall 30-day incidence of MI was 1.1%. We hypothesized that the lower incidence of MI is related to early antiaggregant medical therapy after operation.

At follow-up, the FREEDOM clinical trial and the SYNTAX II clinical trial evidenced an overall incidence of MI of 6% and 2.3%, respectively. In this context, our study evidenced an overall incidence of MI of 2.3%. While our mean population age was 5–7 years older compared to populations of the aforementioned clinical trials, our clinical outcomes further evidenced that patients with diabetes are at high risk for long-term clinical complications.

### 4.6. Incidence of Reoperation

Reoperations and reinterventions are not only a burden for patients, but also for hospitals and healthcare by way of readmissions and increasing costs and use of resources. The incidence of reoperation in patients with diabetes in the current series was 12.3% at 5 years and 23.4% at 10-year follow-up, which is similar to that reported by clinical trials. In this context, the SYNTAX II clinical trial reported an incidence of repeat intervention of 12.9%. In a study analyzing the relationship between diabetes and survival after revascularization in patients with multivessel coronary artery disease at Duke University, Barsness et al. reported a 7% reoperation rate at 5 years after CABG in diabetic patients [17]. Atherosclerosis is a progressive and irreversible disease and is linked with diabetes mellitus through several pathological pathways. Some factors that have been associated with an increased risk of development and progression of atherosclerosis in diabetic patients are dyslipidemia with increased levels of atherogenic LDL, hyperglycemia, oxidative stress, and increased inflammation [22]. Therefore, progression of disease and reoperations/reinterventions become an inevitability.

### 4.7. Clinical Impact of Risk Predictors from Our Study

Significant modifiable risk factors in diabetic patients include peripheral vascular disease (PVD) and chronic obstructive pulmonary disease (COPD) for all-cause mortality, whereas a higher ejection fraction (EF) serves as a protective predictor (Table 5). With respect to MACCE, modifiable predictors include elevated creatinine levels, COPD, and a BMI > 30 kg/m^2^ (Table 5). Our group has previously reported that diabetes is a significant preoperative risk factor in patients with COPD undergoing CABG [23]. Optimal management requires careful patient selection, preoperative optimization of pulmonary function, and specialized postoperative care. Although the impact of COPD varies with disease severity, it consistently represents a major comorbidity that necessitates coordinated cardiology–pulmonology management [19].

PVD is also a major determinant of long-term prognosis in diabetic patients undergoing CABG. Levitt and colleagues [24] demonstrated that survival is significantly influenced by the coexistence of PVD, with diabetic patients exhibiting a mortality rate of 4.4 deaths per 100 person-years—approximately four times higher than that of nondiabetic patients. More recently, a clinical trial published in *The Lancet* by Verma et al. [25] showed that patients with PVD and a history of CABG treated with semaglutide experienced a lower incidence of MACE and adverse events. These findings suggest that appropriate preoperative management of PVD in diabetic patients may reduce MACCE and improve outcomes.

Furthermore, elevated creatinine levels in our cohort were associated with a high hazard ratio for both all-cause mortality and MACCE. Our group has previously shown that diabetes increases the risk of all-cause mortality in patients with elevated creatinine [26]. Additionally, studies by Levitt et al. and Farkouh et al. have demonstrated that chronic kidney disease (CKD) in diabetic patients substantially increases mortality risk following CABG [24,27]. Perioperative medical optimization in patients with CKD and diabetes has been shown to improve clinical outcomes, shorten hospital length of stay, and reduce overall healthcare costs.

## 5. Limitations

This retrospective study was subject to all limitations inherent to a non-randomized study, including potential selection bias. Some of the differences between the diabetic and non-diabetic patient groups would have been mitigated through the use of propensity-matched analysis. However, even the most sophisticated statistical methods often cannot account for all unmeasurable confounders. Additionally, the study period includes a large timeframe (2005–2021), which involves the evolution of many advanced surgical and anesthetic techniques and changes in perioperative care. Furthermore, ours is a single-center study, the results of which cannot be extrapolated to all institutions, and therefore necessitates further validation from multicenter studies. Another important limitation of this clinical study is the lack of a SYNTAX score, which was not recorded in the original database. Our original database lacked the data regarding statin use, antiplatelet regimen, graft patency data, diabetes duration, and modern therapies, including SGLT-2 inhibitors, etc.

## 6. Conclusions

Patients with diabetes undergoing isolated CABG had a significantly higher incidence of all-cause death and MACCE compared to those without diabetes. The presence of diabetes mellitus predicts poorer long-term outcomes following CABG.

Modifiable risk predictors for all-cause mortality and MACCE include COPD, increased creatinine level, and PVD, while non-modifiable predictors include age and an STS score higher than 4%.

## Figures and Tables

**Table 1:** Pre-operative patient characteristics before and after propensity matching.

	Unmatched Patients			Matched Patients		
	No Diabetes *n* = 2852	Diabetes *n* = 2019	*p*-Value	No Diabetes *n* =1589	Diabetes *n* =1589	*p*-Value
Age Years (mean/SD)	71.4 (11.2)	70.5 (10.8)	0.006	71.22 (11.1)	71.43 (10.6)	0.5
Gender			<0.0001			0.7
Female *n* (%)	602 (21.1%)	575 (28.5%)		404 (25.4%)	396 (24.9%)	
Male *n* (%)	2250 (78.9%)	1444 (71.5%)		1185 (75.1%)	1193 (75.0%)	
Race			<0.0001			0.8
White *n* (%)	2563 (89.9%)	1742 (86.3%)		1390 (87.5%)	1399 (88.0%)	
Black or African American *n* (%)	239 (8.4%)	222 (11.0%)		162 (10.2%)	156 (9.8%)	
Other *n* (%)	50 (1.8%)	55 (2.7%)		37 (2.3%)	34 (2.1%)	
STS Risk of Mortality % (median/IQR)	0.84 (0.47–1.8)	1.2 (0.64–2.6)	<0.0001	0.98 (0.51–2.1)	1.1 (0.59–2.37)	0.02
BMI kg/m^2^ (Mean/SD)	28.1 (4.9)	30.9 (11.6)	<0.0001	29.4 (5.35)	29.2 (5.01)	0.1
Obese *n* (%)	850 (29.8%)	1008 (49.9%)	<0.0001	652 (41.0%)	633 (39.8%)	0.4
Creatine Level (Median/IQR)	1 (0.9–1.2)	1 (0.9–1.3)	<0.0001	1 (0.9–1.2)	1 (0.9–1.3)	0.08
Creatinine level < 1.2 mg/dL	2083 (73.0%)	1282 (63.5%)		116 (70.2%)	1056 (66.5%)	
Creatinine level 1.2 to 1.5 mg/dL	588 (20.6%)	432 (21.4%)		343 (21.6%)	350 (22.0%)	
Creatinine level > 1.5 to 2.0 mg/dL	97 (3.4%)	155 (7.7%)		58 (3.7%)	110 (6.9%)	
Creatinine level > 2.0 or on dialysis	84 (3.0%)	150 (7.4%)		72 (4.5%)	73 (4.6%)	
Dialysis *n* (%)	39 (1.4%)	80 (4.0%)	<0.0001	36 (2.3%)	28 (1.8%)	0.2
Smoking *n* (%)	1303 (45.7%)	964 (47.7%)	0.1	757 (47.6%)	762 (47.9%)	0.8
COPD *n* (%)	398 (14.0%)	369 (18.3%)	<0.0001	259 (16.3%)	254 (16.0%)	0.8
Hypertension *n* (%)	2321 (81.4%)	1884 (93.3%)	<0.0001	1461 (91.9%)	1455 (91.6%)	0.6
Dyslipidemia *n* (%)	2462 (86.3%)	1769 (87.6%)	0.1	1375 (86.5%)	1380 (86.8%)	0.7
CBVD *n* (%)	443 (15.5%)	455 (22.5%)	<0.0001	307 (19.3%)	315 (19.8%)	0.7
PVD *n* (%)	327 (11.5%)	395 (19.6%)	<0.0001	251 (15.8%)	251 (15.8%)	1
Liver disease *n* (%)	35 (1.2%)	27 (1.3%)	0.7	25 (1.6%)	19 (1.2%)	0.3
Prior Mediastinal Radiation *n* (%)	23 (0.8%)	22 (1.1%)	0.3	17 (1.1%)	16 (1.0%)	0.8
Previous PCI *n* (%)	997 (35.0%)	821 (40.7%)	<0.0001	612 (38.5%)	629 (39.6%)	0.5
Prior CABG *n* (%)	65 (2.3%)	47 (2.3%)	0.9	36 (2.3%)	41 (2.6%)	0.5
Prior MI *n* (%)	1539 (54.0%)	1185 (58.7%)	0.001	891 (56.1%)	894 (56.3%)	0.9
Prior Valve Surgery *n* (%)	16 (0.6%)	14 (0.7%)	0.5	11 (0.7%)	13 (0.8%)	0.6
Atrial Fibrillation *n* (%)	344 (12.1%)	249 (12.3%)	0.7	210 (13.2%)	202 (12.7%)	0.6
Pre-operative EF (mean/SD) *n* (%)	53.4 (12.7)	51.1 (14.1)	<0.0001	52.4 (13.3)	52.5 (13.5)	0.8
EF *n* (%)			<0.0001			0.233
EF < 25%	99 (3.5%)	108 (5.4%)		70 (4.4%)	61 (3.8%)	
EF 25–50%	1029 (36.1%)	786 (38.9%)		608 (38.3%)	571 (35.9%)	
EF > 50%	1724 (60.4%)	1125 (55.7%)		911 (57.3%)	957 (60.2%)	
Diseased Vessels			<0.0001			0.7
1 *n* (%)	322 (11.3%)	137 (6.8%)		132 (8.3%)	124 (7.8%)	
2 *n* (%)	770 (27.0%)	455 (22.5%)		400 (25.2%)	399 (25.1%)	
3 *n* (%)	1666 (58.4%)	1340 (66.4%)		990 (62.3%)	1009 (63.5%)	
4 *n* (%)	94 (3.3%)	87 (4.3%)		67 (4.2%)	57 (3.6%)	
Left Main Coronary Artery Stenosis > 50% *n* (%)	744 (26.1%)	487 (24.1%)	0.1	392 (24.7%)	402 (25.3%)	0.6
Severe Proximal LAD Lesion > 70% *n* (%)	2373 (83.2%)	1714 (84.9%)	0.1	1336 (84.1%)	1333 (83.9%)	0.8
Insulin *n* (%)	0	1825 (90.4%)	<0.0001	0	1482 (93.2%)	<0.0001
Metformin *n* (%)	0	1025 (50.7%)	<0.0001	0	892 (56.1%)	<0.0001
B-blockers *n* (%)	2125 (74.3%)	1926 (95.3%)	<0.0001	1482 (93.2%)	1476 (92.8%)	0.5
ACE-ARB inhibitors *n* (%)	1254 (43.9%)	1845 (91.3%)	<0.0001	956 (60.1%)	1543 (97.1%)	<0.0001

EF—ejection fraction; IMA—internal mammary artery; LAD—left anterior descending; MI—myocardial infarction; CABG—coronary artery bypass grafting; COPD—chronic obstructive pulmonary disease; HTN—hypertension; PVD—peripheral vascular disease; CBVD—cerebrovascular disease; RIMA—right internal mammary artery; PCI—percutaneous coronary intervention; ACE-ARB—angiotensin converting enzyme–angiotensin receptor blockers.

**Table 2:** Intra-operative outcomes.

	Unmatched			Matched		
	No Diabetes *n* = 2852	Diabetes *n* = 2019	*p*-Value	No Diabetes *n* =1589	Diabetes *n* =1589	*p*-Value
IMA use			<0.0001			0.7
Single IMA *n* (%)	2343 (82.2%)	1890 (93.6%)		1447 (92.9%)	1467 (92.3%)	
IMA + RIMA *n* (%)	457 (16.0%)	90 (4.5%)		81 (5.1%)	89 (5.6%)	
None *n* (%)	52 (1.8%)	39 (1.9%)		31 (1.9%)	33 (2.1%)	
Radial Artery Graft, *n* (%)	547 (19.2%)	346 (17.1%)	0.07	270 (17.0%)	278 (17.5%)	0.7
SVG, *n* (%)	1340 (50.0%)	1107 (54.8%)	<0.0001	797 (49.8%)	842 (53%)	0.1
Number of Grafts (median/IQR)	2 (1–3)	2 (1–3)	0.05	2 (1–3)	2 (1–3)	0.05
Number of Grafts			0.2			0.2
1	1227 (43.0%)	811 (40.2%)		712 (44.8%)	665 (41.8%)	
2	479 (16.8%)	349 (17.3%)		260 (16.4%)	260 (16.4%)	
3	676 (23.7%)	512 (25.4%)		375 (23.6%)	398 (25.0%)	
4	359 (12.6%)	256 (12.7%)		192 (12.1%)	195 (12.3%)	
5+	111 (3.9%)	91 (4.5%)		50 (3.1%)	71 (4.5%)	
Total Arterial CABG *n* (%)	792 (27.8%)	386 (19.1%)	<0.0001	307 (19.3%)	319 (20.1%)	0.5
Multiple Arterial CABG *n* (%)	844 (29.6%)	433 (21.5%)	<0.0001	368 (23.2%)	376 (23.7%)	0.7
On Pump CABG *n* (%)	358 (12.5%)	333 (16.5%)	<0.0001	236 (14.8%)	254 (16.0%)	0.3
Priority of Surgery (%)			0.02			0.3
Elective	1574 (55.2%)	1037 (51.4%)		860 (54.1%)	827 (52.0%)	
Urgent	1251 (43.9%)	966 (47.9%)		712 (44.8%)	748 (47.1%)	
Emergent	27 (1.0%)	16 (0.8%)		17 (1.1%)	14 (0.9%)	
Time in OR (Hours) (Median/IQR)	5.8 (5.1–6.7)	5.8 (5.2–6.7)	0.1	5.8 (5.1–6.7)	5.7 (5.1–6.7)	0.2
All types of blood product transfusions *n* (%)	452 (15.9%)	446 (22.1%)	<0.0001	287 (18.1%)	338 (21.3%)	**0.02**
RBC Units *n* (%)	368 (12.9%)	404 (20.0%)	<0.0001	238 (15.0%)	299 (18.8%)	**0.004**
Cryoprecipitate Units *n* (%)	103 (3.6%)	84 (4.2%)	0.3	66 (4.1%)	65 (4.1%)	0.9
Platelet Units *n* (%)	182 (6.4%)	159 (7.9%)	0.04	117 (7.4%)	121 (7.6%)	0.7
FFP Units *n* (%)	47 (1.7%)	60 (3.0%)	0.002	26 (1.6%)	44 (2.8%)	**0.02**
Extubated in OR *n* (%)	2290 (80.3%)	1465 (72.6%)	<0.0001	1217 (76.6%)	1200 (75.5%)	0.4

IMA—internal mammary artery; SVG—saphenous venous graft; CABG—coronary artery bypass grafting; FFP—fresh frozen plasma; RBC—red blood cells; OR—operating room.

**Table 3:** Post-operative outcomes.

	Unmatched Patients			Matched Patients		
	No Diabetes *n* = 2852	Diabetes *n* = 2019	*p*-Value	No Diabetes *n* =1589	Diabetes *n* =1589	*p*-Value
Total ICU (Hours) (Median/IQR)	43.6 (24.7–71.8)	47.0 (25.5–87.6)	<0.0001	44.7 (25.1–72.3)	45.6 (25.0–75.6)	0.08
Total LOS (Days) (Median/IQR)	5 (4–6)	5 (4–7)	<0.0001	5 (4–7)	5 (4–7)	**0.0005**
All types of Blood Products Transfusion *n* (%)	851 (29.8%)	689 (34.1%)	0.002	503 (31.7%)	515 (32.4%)	0.6
RBC Units *n* (%)	826 (29.0%)	673 (33.3%)	0.001	485 (30.5%)	501 (31.5%)	0.5
Cryoprecipitate Units *n* (%)	139 (4.9%)	61 (3.0%)	0.001	76 (4.8%)	50 (3.15%)	**0.01**
Platelet Units *n* (%)	188 (6.6%)	88 (4.4%)	0.001	101 (6.4%)	66 (4.1%)	**0.004**
FFP Units *n* (%)	107 (3.8%)	69 (3.4%)	0.5	65 (4.1%)	56 (3.5%)	0.4
Stroke *n* (%)	6 (0.2%)	22 (1.1%)	<0.0001	5 (0.3%)	17 (1.1%)	**0.01**
Superficial Sternal Wound Infection *n* (%)	4 (0.1%)	13 (0.6%)	0.003	2 (0.1%)	9 (0.6%)	**0.03**
Deep Sternal Infection *n* (%)	5 (0.2%)	11 (0.5%)	0.02	5 (0.3%)	4 (0.2%)	0.7
Reoperation for Bleeding *n* (%)	34 (1.2%)	13 (0.6%)	0.05	18 (1.1%)	11 (0.7)	0.1
Prolonged Ventilation > 24 h *n* (%)	87 (3.1%)	116 (5.8%)	<0.0001	63 (4.0%)	80 (5.0%)	0.1
Renal Failure *n* (%)	39 (1.4%)	53 (2.6%)	0.001	24 (1.5%)	30 (1.9%)	0.4
New Dialysis *n* (%)	9 (0.3%)	16 (0.8%)	0.02	6 (0.4%)	9 (0.6%)	0.4
New Atrial Fibrillation *n* (%)	628 (22.0%)	468 (23.2%)	0.3	365 (23%)	376 (24%)	0.6
30 Day Readmission *n* (%)	210 (7.4%)	181 (9.0%)	0.04	117 (7.4%)	136 (8.6%)	0.2
30-day mortality *n* (%)	22 (0.8%)	28 (1.4%)	0.036	17 (1.1%)	17 (1.1%)	1

LOS—length of stay; FFP—fresh frozen plasma; RBC—red blood cells; ICU—intensive care unit.

**Table 4:** Cumulative long-term outcomes.

	Unmatched Patients			Matched Patients		
	No Diabetes *n* = 2852	Diabetes *n* = 2019	*p*-Value	No Diabetes *n* =1589	Diabetes *n* =1589	*p*-Value
All-cause Mortality *n* (%)	267 (9.4%)	296 (14.7%)	<0.0001	169 (10.6%)	215 (13.5%)	**0.013**
MACCE *n* (%)	356 (12.5%)	363 (18.0%)	<0.0001	228 (14.4%)	264 (16.6%)	**0.04**
Non-Fatal Stroke *n* (%)	55 (1.9%)	42 (2.1%)	0.7	35 (2.2%)	34 (2.1%)	0.9
Non-Fatal MI *n* (%)	62 (2.2%)	57 (2.8%)	0.1	44 (2.8%)	37 (2.3%)	0.4
Reoperation *n* (%)	322 (11.3%)	246 (12.2%)	0.3	182 (11.5%)	189 (11.9%)	0.6
Angina *n* (%)	303 (10.6%)	231 (11.4%)	0.3	165 (10.4%)	182 (11.5%)	0.3

MACCE—all-cause death, MI, stroke, and reoperation; MI—myocardial infarction.

**Table 5:** Independent predictors of long-term outcomes in diabetic vs. non-diabetic patients.

All-Cause Death	All Patients HR (95% CI)	Diabetes HR (95% CI)	No Diabetes HR (95% CI)
Diabetes	1.4 (1.2, 1.7)		
Age > 75 years	1.02 (1.01, 1.03)		**1.04 (1.01, 1.05)**
STS-PROM risk score > 4%	1.04 (1.02, 1.06)	1.06 (1.03, 1.08)	1.03 (1.01, 1.1)
Creatinine level			
1.2 to 1.5 mg/dL	1.3 (1.1, 1.6)	**1.4 (1.1, 1.9)**	
1.5 to 2.0 mg/dL	1.8 (1.3, 2.5)	**2.1 (1.4, 3.2)**	
>2.0 mg/dL or on dialysis	3.3 (2.5, 4.4)	3.1 (2.1, 4.5)	3.7 (2.4, 6.0)
Prior Valve Surgery			**3.3 (1.2, 9.3)**
Prior MI	1.3 (1.05, 1.5)		**1.6 (1.2, 2.1)**
PVD	1.4 (1.2, 1.7)	1.4 (1.04, 1.8)	1.6 (1.2, 2.2)
Atrial Fibrillation			**1.5 (1.1, 2.1)**
CBVD	1.2 (1.01, 1.5)		
COPD	1.4 (1.2, 1.7)	1.4 (1.04, 1.8)	1.7 (1.3, 2.2)
BMI > 30 kg/m^2^			0.96 (0.93, 0.99)
Previous PCI			
Preoperative EF	0.98 (0.98, 0.99)	0.98 (0.97, 0.99)	
**MACCE**	**HR (95% CI)**	**HR (95% CI)**	**HR (95% CI)**
Diabetes	1.2 (1.03, 1.3)		
Age > 75 years	0.99 (0.98, 0.99)	0.98 (0.98, 0.99)	
STS-PROM risk score > 4%	1.06 (1.04, 1.07)	1.04 (1.02, 1.07)	1.06 (1.05, 1.08)
Creatinine level mg/dL			
1.5 to 2.0 mg/dL	1.5 (1.1, 1.9)	**1.7 (1.3, 2.4)**	
>2.0 mg/dL or on dialysis	2.1 (1.6, 2.6)	2.1 (1.5, 2.9)	2.1 (1.5, 3.0)
PVD	1.2 (1.02, 1.4)		**1.3 (1.03, 1.6)**
COPD	1.2 (1.1, 1.4)		**1.3 (1.01, 1.6)**
CBVD	1.3 (1.1, 1.5)	1.3 (1.04, 1.6)	1.3 (1.04, 1.6)
Prior Valve Surgery			**2.7 (1.2, 6.3)**
BMI > 30 kg/m^2^	1.0 (1.0, 1.01)	**1.06 (1.03, 1.1)**	
Four Diseased Vessels (Ref = 1)	1.5 (1.04, 2.3)		
Prior Mediastinal Radiation	2.2 (1.3, 3.9)	**2.6 (1.3, 5.3)**	
Black Race (Ref = White)			**1.5 (1.1, 1.9)**
Previous PCI	1.4 (1.2, 1.6)	1.3 (1.1, 1.6)	1.4 (1.2, 1.7)
**STROKE**	HR (95% CI)	HR (95% CI)	HR (95% CI)
Diabetes	0.99 (0.7, 1.5)		
Prior MI	1.7 (1.1, 2.7)		
Black Race (Ref = White)	1.9 (1.1, 3.4)	**3.2 (1.6, 6.7)**	
CBVD	2.2 (1.4, 3.5)	2.7 (1.4, 5.0)	2.0 (1.04, 3.9)
Four Diseased Vessels (Ref = 1)	6.1 (1.5, 25.2)		
Preoperative EF	1.02 (1.01, 1.04)	**1.03 (1.01, 1.06)**	
**MI**	**HR (95% CI)**	**HR (95% CI)**	**HR (95% CI)**
Diabetes	1.03 (0.7, 1.5)		
Age > 75 years	0.98 (0.97, 0.99)		
BMI > 30 kg/m^2^	1.01 (1.01, 1.01)	1.01 (1.01, 1.01)	1.08 (1.03, 1.14)
Previous PCI	2.0 (1.4, 2.9)	2.0 (1.2, 3.4)	2.1 (1.3, 3.5)
Black Race (Ref = White)	1.8 (1.1, 2.9)		**2.0 (1.02, 3.9)**
Hypertension	2.1 (1.1, 4.2)		
CVBD	1.7 (1.1, 2.6)		
Three Diseased Vessels (Ref = 1)	2.8 (1.03, 7.8)		
STS-PROM risk score > 4%			1.03 (1.01, 1.08)
Left Main Coronary Artery Stenosis > 50%			1.8 (1.1, 2.9)
**REOPERATION**	**HR (95% CI)**	**HR (95% CI)**	**HR (95% CI)**
Diabetes	1.1 (0.9, 1.3)		
BMI > 30 kg/m^2^	1.0 (1.0, 1.01)	1.01 (1.0, 1.01)	1.03 (1.01, 1.06)
Age > 75 years	0.98 (0.98, 0.99)	0.98 (0.97, 0.99)	0.98 (0.98, 0.99)
Female (Ref = Male)	1.3 (1.1, 1.6)		**1.3 (1.01, 1.7)**
Previous PCI	1.5 (1.3, 1.8)	1.6 (1.2, 2.0)	1.4 (1.1, 1.8)
Left Main Stenosis >50%	1.2 (1.02, 1.5)		**1.4 (1.1, 1.8)**
Prior Mediastinal Radiation		**3.1 (1.3, 7.0)**	
Preoperative EF	1.01 (1.0, 1.02)	1.02 (1.01, 1.03)	1.01 (1.01, 1.02)
Black Race (Ref: White)			**1.6 (1.1, 2.2)**
Severe Proximal LAD Lesion > 70%		**1.6 (1.1, 2.4)**	

MI—myocardial infarction; PVD—peripheral vascular disease; COPD—chronic obstructive pulmonary disease; CBVD—cerebrovascular disease; BMI—body mass index; PCI—percutaneous coronary intervention.

## Data Availability

The data that support the findings of this study are available upon reasonable request to S. Sicouri, pending institutional approval.

## Supplementary Materials

The following supporting information can be downloaded at: https://www.mdpi.com/article/10.3390/medicina62010071/s1, Table S1. Observed cumulative HR incidence of long-term outcomes.

## Abbreviations

The following abbreviations are used in this manuscript:

CABG	coronary artery bypass grafting
CAD	coronary artery disease
CKD	chronic kidney disease
COPD	chronic obstructive pulmonary disease
PVD	peripheral vascular disease
OR	operating room
ICU	intensive care unit
BMI	body mass index
SVG	saphenous venous grafting
IMA	internal mammary artery
MI	myocardial infarction
CBVD	cerebrovascular disease
RBC	red blood cells
FFP	fresh frozen plasma
PCI	percutaneous coronary intervention
MACCE	major cardiovascular and cerebrovascular events
