# Supplementary material for: Impact of Periprocedural Risk Predictors on Long-Term Outcomes in Patients with Diabetes Undergoing Coronary Artery Bypass Grafting
Source: Medicina (Kaunas). 2025 Dec 29;62(1):71. doi: 10.3390/medicina62010071 (PMC12843129; doi:10.3390/medicina62010071)
Supplement: Supplementary file 1 [file medicina-62-00071-s001.zip › medicina-3906842-Table S1.pdf]

Table S1. Observed cumulative HR incidence of long-term outcomes.

Long-Term Outcomes	No Diabetes N = 1589	Diabetes N = 1589	*p*-Value
**All-Cause Mortality**			
1 year n (%)	29 (1.8%)	44 (2.8%)	0.07
2 years n (%)	41 (2.6%)	63 (4.0%)	**0.02**
5 years n (%)	79 (5.0%)	111 (7.0%)	**0.01**
10 years n (%)	143 (9.1%)	181 (11.6%)	**0.02**
**MACCE**			
1 year n (%)	33 (2.1%)	54 (3.4%)	**0.02**
2 years n (%)	50 (3.2%)	75 (4.7%)	**0.02**
5 years n (%)	112 (7.1%)	140 (8.8%)	**0.04**
10 years n (%)	196 (12.3%)	225 (14.2%)	0.1
**Stroke**			
1 year n (%)	0 (0.0%)	7 (0.4%)	**0.008**
2 years n (%)	3 (0.2%)	7 (0.4%)	0.2
5 years n (%)	13 (0.8%)	19 (1.2%)	0.2
10 years n (%)	29 (1.8%)	27 (1.7%)	0.7
**Myocardial Infarction**			
1 year n (%)	4 (0.2%)	8 (0.5%)	0.2
2 years n (%)	7 (0.4%)	10 (0.6%)	0.4
5 years n (%)	23 (1.4%)	20 (1.3%)	0.6
10 years n (%)	39 (2.4%)	34 (2.1%)	0.5
**Reoperation**			
1 year n (%)	23 (1.4%)	28 (1.8%)	0.4
2 years n (%)	51 (3.2%)	55 (3.5%)	0.6
5 years n (%)	105 (6.6%)	111 (7.0%)	0.6
10 years n (%)	169 (10.6%)	177 (11.1%)	0.6
**Angina**			
1 year n (%)	24 (1.7%)	30 (2.1%)	0.3
2 years n (%)	56 (3.5%)	62 (3.9%)	0.5
5 years n (%)	100 (6.3%)	116 (7.3%)	0.2
10 years n (%)	150 (9.4%)	168 (10.6%)	0.2

Long-Term Outcomes	Unweighted HR (95% CI)	*p*-value	Weighted HR (95% CI)	*p*-Value
**All-Cause Mortality**				
1 year	1.5 (0.9, 2.4)	0.08	1.7 (1.1, 2.8)	**0.02**
2 years	1.5 (1.03, 2.3)	0.03	1.7 (1.1, 2.5)	**0.01**
5 years	1.4 (1.1, 1.9)	0.01	1.5 (1.1, 2.0)	**0.009**
10 years	1.3 (1.03, 1.6)	0.02	1.3 (1.04, 1.6)	**0.02**
**MACCE**				
1 year	1.6 (1.1, 2.5)	0.02	1.9 (1.2, 3.0)	**0.005**
2 years	1.5 (1.05, 2.2)	0.02	1.7 (1.1, 2.4)	**0.008**
5 years	1.3 (0.9, 1.6)	0.05	1.4 (1.05, 1.8)	**0.01**
10 years	1.2 (0.96, 1.4)	0.1	1.2 (0.99, 1.5)	0.06
**Stroke**				
1 years	N/A		N/A	
2 years	2.3 (0.6, 9.0)	0.2	2.4 (0.6, 9.2)	0.2

5 years	1.5 (0.7, 3.0)	0.2	1.5 (0.7, 3.04)	0.2
10 years	0.9 (0.6, 1.6)	0.8	0.9 (0.5, 1.6)	0.8
**Myocardial Infarction**				
1 year	2.0 (0.6, 6.6)	0.2	2.0 (0.6, 6.7)	0.2
2 years	1.4 (0.5, 3.8)	0.4	1.5 (0.6, 3.9)	0.4
5 years	0.9 (0.5, 1.6)	0.7	0.9 (0.5, 1.7)	0.7
10 years	0.9 (0.6, 1.4)	0.6	0.9 (0.6, 1.4)	0.6
**Reoperation**				
1 years	1.2 (0.7, 2.1)	0.4	1.2 (0.7, 2.1)	0.4
2 years	1.1 (0.7, 1.6)	0.6	1.1 (0.7, 1.6)	0.6
5 years	1.1 (0.8, 1.4)	0.5	1.1 (0.8, 1.4)	0.4
10 years	1.1 (0.9, 1.3)	0.5	1.1 (0.9, 1.3)	0.4
**Angina**				
1 year	1.3 (0.7, 2.1)	0.4	1.2 (0.7, 2.1)	0.4
2 years	1.1 (0.8, 1.6)	0.5	1.1 (0.8, 1.6)	0.5
5 years	1.2 (0.9, 1.6)	0.1	1.2 (0.9, 1.6)	0.1
10 years	1.1 (0.9, 1.4)	0.2	1.2 (0.9, 1.5)	0.2
